# Microfilaria in cytology smears from upper arm swelling

**DOI:** 10.4103/0970-9371.73307

**Published:** 2010-10

**Authors:** Ruma Pahwa, Vivek Mohan Arora

**Affiliations:** Department of Pathology, Dr. Baba Saheb Ambedkar Government Hospital, Delhi, India; 1 Department of Microbiology, Dr. Baba Saheb Ambedkar Government Hospital, Delhi, India

Sir,

Filariasis is endemic in tropical countries such as India, China, Indonesia, parts of Asia and Africa.[1] The adult filarial worms reside in the lymphatic system from where the gravid female releases large number of microfilaria which may pass through the thoracic duct and pulmonary capillaries into the peripheral blood, and occasionally, microfilaraemia may be found in the infected patient. Microfilaria have been reported in variable locations like epididymis,[2] thyroid,[3] breast[4] and in variable specimens like bronchial washings, urine[5] and ovarian fluids.[6] Many of the cases reported have been incidental findings on fine needle aspiration cytology (FNAC) examination. Microfilaria of *Wuchereria bancrofti* are identified morphologically by the presence of hyaline sheath, length of cephalic space and presence of nuclei from head to tail, with tip free from the nuclei. It is common to find microfilaria in axillary lymph nodes;[7] however, to find them in a cystic swelling in the upper arm is very rare.

**Figure 1:**
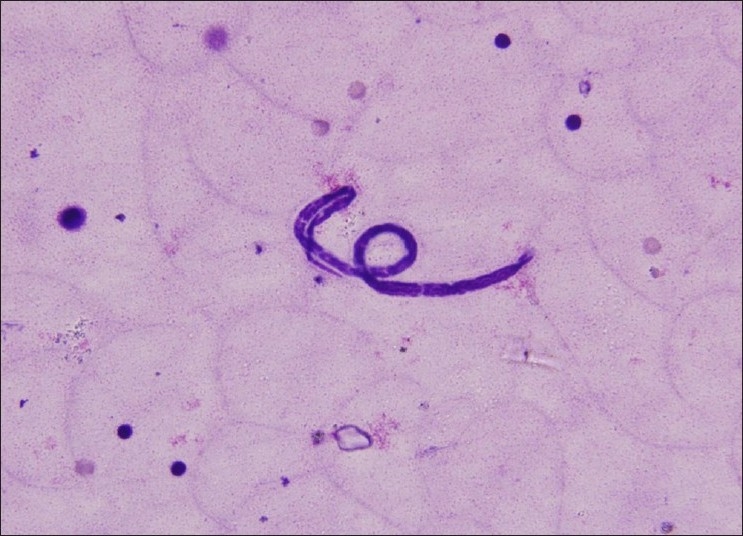
FNAC smear shows microfilaria of *Wuchereria bancrofti* (tail tip free of nuclei) in a clear background (Giemsa, ×400)

We present an interesting case of an 18-year-old boy who presented with a cystic swelling on the medial aspect of the upper arm of 1 week duration, and was referred to the pathology department for FNAC. The swelling measured 2.5 × 1.0 cm and FNAC yielded straw colored fluid, Giemsa stained smears prepared from which showed numerous microfilaria of *Wuchereria bancrofti* in a clear background [Figure 1]. On further investigation, eosinophilia and microfilaraemia were found in the peripheral blood film. Prompt therapy was started which led to the resolution of the infection.

In conclusion, we emphasize the importance to consider filariasis in differential diagnosis in unusual cystic swellings, especially in endemic countries.
